# The Sybtraps: Control of Synaptobrevin Traffic by Synaptophysin, α-Synuclein and AP-180

**DOI:** 10.1111/tra.12140

**Published:** 2013-12-16

**Authors:** Sarah L Gordon, Michael A Cousin

**Affiliations:** Membrane Biology Group, Centre for Integrative Physiology, George Square, University of EdinburghScotland, EH8 9XD, UK

**Keywords:** AP-180, endocytosis, presynapse, synaptobrevin, synaptophysin, vesicle, α-synuclein

## Abstract

Synaptobrevin II (sybII) is a key fusogenic molecule on synaptic vesicles (SVs) therefore the active maintenance of both its conformation and location in sufficient numbers on this organelle is critical in both mediating and sustaining neurotransmitter release. Recently three proteins have been identified having key roles in the presentation, trafficking and retrieval of sybII during the fusion and endocytosis of SVs. The nerve terminal protein α-synuclein catalyses sybII entry into SNARE complexes, whereas the monomeric adaptor protein AP-180 is required for sybII retrieval during SV endocytosis. Overarching these events is the tetraspan SV protein synaptophysin, which is a major sybII interaction partner on the SV. This review will evaluate recent studies to propose working models for the control of sybII traffic by synaptophysin and other Sybtraps (sybII trafficking partners) and suggest how dysfunction in sybII traffic may contribute to human disease.

The regulated fusion of synaptic vesicles (SVs) on action potential invasion of central nerve terminals mediates neurotransmitter release. The subsequent retrieval and recycling of SVs after fusion is essential for the maintenance of neurotransmission. The SV is a highly organized molecular machine; therefore the mechanisms which control the sequestering of SV cargo from the plasma membrane during endocytosis are critical for generating functional organelles that are capable of responding to neuronal activity with high temporal and spatial precision. Cargo clustering and selection occurs at the plasma membrane during SV formation, via the coordinated recruitment of the classical adaptor protein complex AP-2 1,2. Some cargo proteins contain classical recognition motifs for the μ2 subunit of AP-2 2, however some do not, suggesting interactions with other molecules must facilitate their sequestration into SVs. Recently a number of monomeric adaptor proteins have been identified that interact with specific SV cargo to assist their retrieval  3–5. However, even these events may not be sufficient to ensure the maximal efficiency of SV cargo retrieval. Instead it seems that interactions between cargo molecules themselves may also be required to facilitate their clustering and incorporation into nascent SVs 6.

## Synaptobrevin II and Synaptophysin: Two Major SV Proteins

Synaptobrevin II (sybII) and synaptophysin are the two most abundant SV cargo proteins, with approximately 70 and 30 copies, respectively per SV 7. SybII is a single pass transmembrane SV protein that has an established essential role in evoked neurotransmitter release 8. It has a relatively short cytoplasmic N-terminus comprised of a proline rich region followed by a stretch of amino acids called a SNARE (soluble NSF-attachment protein receptor) motif which forms a coiled coil 9. SNARE motifs are also found in the plasma membrane proteins syntaxin and SNAP-25 (synaptosomal-associated protein, 25 kDa) 10,11. It is the progressive association of four SNARE motifs (2 from SNAP-25 and 1 each from sybII and syntaxin) that firstly renders SVs competent for fusion 12 and subsequently drives exocytosis, an event tightly coupled to the influx of calcium into the nerve terminal via the calcium sensor synaptotagmin I 8.

Crosslinking experiments in neurons revealed that outside of the SNARE complex sybII exists in a combination of monomers and dimers 13–15. SybII homodimerization is dependent on specific residues within its transmembrane domain 16–19, however, disruption of dimerization does not impact on its SNARE-dependent fusion activity 19. The affinity of these transmembrane interactions is reported to be weak 20,21 but is promoted by high concentrations of sybII molecules and cholesterol, both of which are found in the local SV environment 22. Thus the role of sybII dimerization is still undetermined, but is unlikely to mediate its primary function in SV fusion.

In contrast to sybII, synaptophysin is a protein which until very recently had no defined function in the SV life cycle. It is a tetraspan (four transmembrane domain) SV protein, with cytoplasmic N- and C-termini. It is a glycoprotein, with glycosylation essential for its targeting to SVs on its exit from the Golgi apparatus 23. The synaptophysin C-terminus contains a series of pentapeptide repeats, all initiated by a tyrosine residue and it is the most heavily tyrosine phosphorylated protein on the SV 24. Synaptophysin also forms homomultimers in the SV membrane 25, with crystal structures suggesting it forms a basket-like structure with a central pore, similar to gap junctions formed by connexin molecules 26. This led to suggestions that it may act as either a SV fusion pore 27, or as a vesicular ion channel 25,28,29, however, definitive evidence for such roles is still absent.

## Synaptophysin and sybII – Interaction Partners on the SV

**Figure 1:**
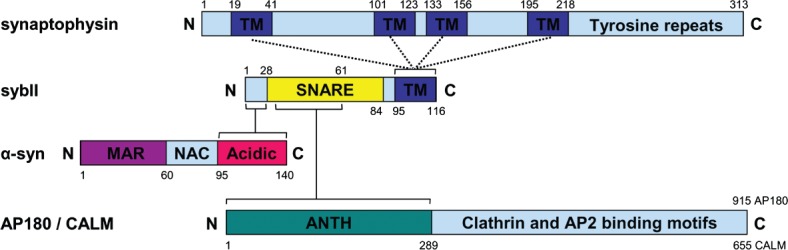
The Sybtraps: sybII trafficking partners Three unrelated proteins interact at separate sites on sybII to control its trafficking and conformation. The integral SV protein synaptophysin is postulated to share a transmembrane (TM) domain interaction with sybII, leaving its cytoplasmic C-terminus (with numerous tyrosine repeats) free to associate with other endocytic molecules. The SV-associated protein α-synuclein interacts with the extreme N-terminus of sybII via its acidic C-terminus. It also binds lipid membranes via its membrane associated region (MAR) on its N-terminus inducing formation of an α-helix, whereas its non-amyloid component (NAC) domain mediates its aggregation. The monomeric endocytic adaptors AP180 and CALM interact with the sybII SNARE motif via their ANTH domains. They can also recruit both clathrin and the AP-2 adaptor complex via multiple interaction motifs on the remaining protein. Numbers indicate amino acid primary sequence.

In addition to being the two most abundant SV proteins, sybII and synaptophysin also form a complex together 13,15,30,31. The complex is thought to be mediated by the transmembrane regions of the two proteins (Figure 1), since the interaction is retained even after removal of their respective cytosolic domains 30,32–34. However, a peptide encompassing the N-terminal 32 amino acids of sybII does dissociate this complex on purified SVs 15, suggesting that the N-terminus of sybII may facilitate complex formation in some manner.

The functional role of the sybII–synaptophysin complex has been investigated by a number of groups. It was established from an early stage that the interaction of sybII with synaptophysin was mutually exclusive to its interaction with other SNARE proteins 13,14. Since up to 25% of sybII can be associated with synaptophysin at any one time 31, this suggested that synaptophysin may control the availability of sybII for SV fusion reactions. In support, FRET analysis of sybII and synaptophysin expressed in central neurons demonstrated that the proteins dissociate on stimulation 35 an observation largely supported by biochemical studies in nerve terminal lysates, which suggested a potential calcium-dependence to this event 36–38. However, the premise that synaptophysin restricts entry of sybII into SNARE complexes is at odds with studies showing sybII freely dissociates from synaptophysin when SVs are exposed to exogenous syntaxin and SNAP-25 39. Furthermore cross-linking studies in intact isolated nerve terminals demonstrated large increases in the sybII–synaptophysin interaction after a number of independent stimulation protocols 31. The timescale of these latter experiments was consistent with an increase in the sybII–synaptophysin complex after completion of exocytosis, suggesting an alternate function for their interaction other than controlling sybII entry into the SNARE complex.

## Synaptophysin Is Required for Efficient sybII Retrieval During Endocytosis

The sybII–synaptophysin interaction has also been proposed to mediate targeting of sybII to SVs. Studies in either heterologous expression systems or primary neuronal culture demonstrated that the overexpression of sybII resulted in either its increased expression at the plasma membrane or mislocalization from nerve terminals 33,35. Both effects were rescued by co-expression of synaptophysin. SybII exited the Golgi in both of these systems, however, suggesting synaptophysin is not required for its chaperoning to SVs *per se*.

The fact that synaptophysin can rescue mistargetted exogenous sybII and that it displayed an increased association with sybII following stimulation suggests that synaptophysin may participate in sybII retrieval during SV endocytosis. In agreement, synaptophysin knockout neurons in culture display a de-enrichment of endogenous sybII from their nerve terminals, and a stranding of the genetic reporter sybII-pHluorin on their plasma membrane 40. Critically, the retrieval of sybII-pHluorin was greatly retarded during SV endocytosis in synaptophysin knockout neurons, an event fully rescued by the expression of wild-type synaptophysin 40. This pronounced defect in retrieval was specific for sybII, since genetic reporters for other SV cargo proteins did not display the same deficiency. Thus, synaptophysin plays a critical and specific role during SV recycling, to facilitate the retrieval of sybII during SV endocytosis.

## Impact of Inefficient sybII Retrieval on SV Recycling

The profound defect in sybII retrieval in synaptophysin knockout mice suggests that there could be subsidiary consequences for SV recycling. The most obvious would be a perturbation in neurotransmitter release, due to a lack of sybII on SVs. However, original studies in synaptophysin knockout mice found no obvious defects in evoked neurotransmission 41. Moreover, the rate of SV exocytosis in synaptophysin knockout neurons was no different to wild-type when monitored using either genetic reporters 42 or lipophilic dyes 40. A potential explanation for the lack of a clear effect on neurotransmitter release is provided by recent evidence determining the number of SNARE complexes required for SV fusion. Multiple groups have estimated that only 1–3 SNARE complexes are required for this event 43–46. In support of this, the copy number of sybII (and synaptophysin) across SVs is variable 47, suggesting that SVs can still operate normally even with twofold differences in their sybII content. Therefore it is likely that even a large reduction in sybII molecules on synaptophysin knockout SVs would still provide sufficient capacity for efficient neurotransmission. One exception would be during repeated neuronal activity, however, where continual recycling could result in SVs becoming dangerously low on fusogenic sybII molecules. However, this specific scenario has not yet been tested in synaptophysin knockout mice.

Ablation of sybII expression at the genomic level results in a slowing in the rate of SV endocytosis 48 in addition to its profound inhibition on evoked SV fusion 49,50. This slowing of SV endocytosis was also seen after knockdown of sybII expression with small hairpin RNA (shRNA) in typical small central nerve terminals 51 or after its digestion with the clostridial neurotoxin tetanus toxin in atypical large nerve terminals 52,53. Thus the absence of sybII retards the speed of SV endocytosis. This means a potential downstream consequence of inefficient sybII retrieval may be an indirect impact on the kinetics of SV endocytosis. In support, two independent studies demonstrated a small but significant slowing in the retrieval of three distinct SV cargo (synaptotagmin I, vGLUT and SV2A) in primary cultures of synaptophysin knockout neurons 40,42. However, dysfunctional sybII retrieval in synaptophysin knockout neurons has recently been functionally separated from effects on endocytic rate 54. In this study a synaptophysin mutant associated with X-linked intellectual disability (XLID) fully rescued endocytosis kinetics but not sybII retrieval in knockout neurons. Thus inefficient sybII retrieval does not impact on the kinetics of SV endocytosis, nor does it have an acute impact on exocytosis. Instead, inefficient sybII retrieval may result in a long-term loss in the ability of neurons to maintain the fidelity of neurotransmission.

## Other sybII Trafficking Partners (Sybtraps) Are Required During SV Recycling

SybII has no canonical endocytosis motifs to mark it for recognition and internalization via classical adaptor protein complexes such as AP-2. Instead its retrieval is mediated by the monomeric adaptor protein AP-180. This requirement was first suggested in *Caenorhabditis elegans*, where mutants of the AP-180 homolog Unc-11 resulted in increased plasma membrane sybII 55. Furthermore either knockdown or overexpression of the AP-180 homologue CALM (clathrin assembly lymphoid myeloid leukaemia) in a heterologus expression system reciprocally controlled plasma membrane levels of exogenously expressed sybII 56. Finally, knockdown of AP-180 or CALM in primary neuronal culture caused both an increase in the surface fraction of exogenously expressed sybII and its de-enrichment from nerve terminals 5, in a very similar manner to that seen in synaptophysin knockout neurons. The interaction site for AP-180 and sybII has been mapped. The N-terminal portion of the sybII SNARE motif interacts with the ANTH (AP-180 N-terminal homology) domain of either AP-180 or CALM 5 (Figure 1). Mutagenesis of key residues within the AP-180 binding site of the SNARE motif resulted in increased or decreased sybII interactions with AP-180, paralleling a reduced or increased expression at the plasma membrane. Thus both AP-180/CALM and synaptophysin are required for the efficient retrieval of sybII during SV endocytosis.

Another recently identified sybII trafficking molecule is the SV-associated protein α-synuclein. α-Synuclein is a small nerve terminal-enriched soluble protein with an N-terminus that forms an amphipathic helix when bound to membrane 57. SybII interacts with α-synuclein on SVs and unlike synaptophysin remains bound to sybII even after its incorporation into the SNARE complex 58. The C-terminal acidic domain of α-synuclein binds to the extreme N-terminal 28 amino acids of sybII, which are not involved in SNARE complex formation 58,59 (Figure 1). It has been proposed that α-synuclein acts as a non-classical chaperone, by catalyzing the formation of SNARE complexes 58,59. However, α-synuclein knockout mice display no defects in SV exocytosis 60,61, indicating the protein plays a facilitatory, rather than requisite role. The association of α-synuclein with sybII should not preclude binding of synaptophysin, since they are proposed to associate at different sites on the protein. However, the sybII–synaptophysin interaction can be disrupted by peptides encompassing the α-synuclein binding site 15, suggesting their interactions with sybII may influence each other.

Research over the past few years have therefore identified a number of proteins essential for sybII traffic. Due to their discrete and complementary function in facilitating the movement of this key molecule, we have termed them Sybtraps.

## Coordination of sybII Trafficking by Sybtraps

**Figure 2:**
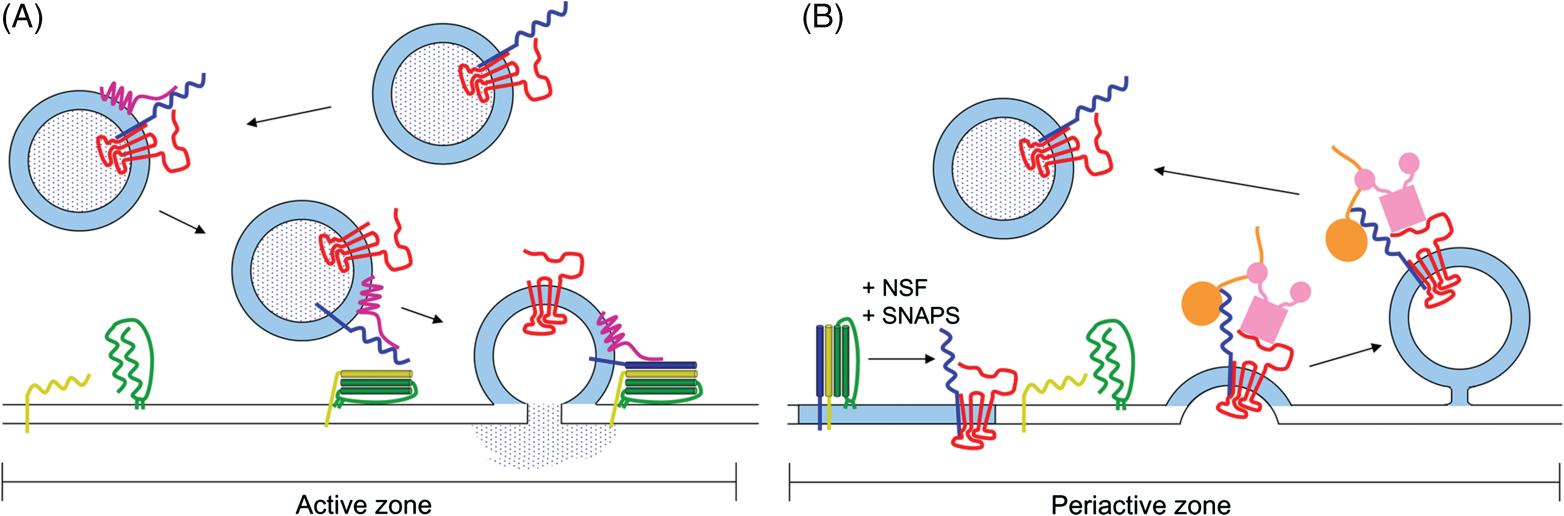
Sybtrap control of sybII during SV recycling A) SybII (blue) is complexed with synaptophysin (red) on the synaptic vesicle in resting nerve terminals via their transmembrane domains. At some point before entry of sybII into the SNARE complex, α-synuclein (purple) interacts with the N-terminus of sybII via its acidic C-terminus. α-Synuclein facilitates the assembly of SNARE complexes between sybII and plasma membrane syntaxin (green)/SNAP-25 (yellow) dimers. Entry of sybII into the *trans*-SNARE complex displaces synaptophysin from sybII. α-Synuclein remains associated with the assembled SNARE complex until synaptic vesicle fusion is triggered. B) After synaptic vesicle fusion, the *cis*-SNARE complex is broken apart by the combined action of N-ethylmaleimide-sensitive factor (NSF) and soluble NSF-attachment proteins (SNAPs). Free monomeric sybII in the plasma membrane is prevented from re-entering futile *cis*-SNARE complexes via a shared transmembrane domain interaction with synaptophysin. This association retains sybII in a ‘retrieval competent’ conformation, where its SNARE motif is accessible to the ANTH domain of the monomeric adaptor AP180 (orange). The avidity of this retrieval complex may be increased further via recruitment of AP-2 (pink) by the tyrosine motifs on the C-terminus of synaptophysin and further still by the subsequent interaction between AP180 and AP-2.

The primary role of the Sybtraps is to ensure that sybII is delivered to the exact location in the correct conformation with high temporal precision. These events are likely to be pivotal in ensuring the high fidelity of neurotransmission. There is now sufficient experimental evidence from multiple independent studies to generate a working model that explains the co-ordinated trafficking of sybII by Sybtraps (Figure 2).

In resting nerve terminals sybII is complexed with synaptophysin on the SV. It is possible that sybII may also simultaneously bind α-synuclein, since both synaptophysin and α-synuclein interact with sybII at independent sites. However, as mentioned above, peptides encompassing the α-synuclein interaction site displace synaptophysin from sybII 15 suggesting they may form mutually exclusive complexes. Regardless, α-synuclein catalyses the formation of *trans*-SNARE complexes by presenting vesicular sybII in the optimal conformation to interact with the plasma membrane SNAP-25/syntaxin1A dimer 58,59. Simultaneously, the formation of the SNARE complex dissociates synaptophysin from sybII. The mechanism of displacement is still unknown, but one possibility is that it is instigated by the engagement of sybII by α-synuclein. Evoked SV fusion then occurs, dependent on the activity-dependent influx of calcium into the nerve terminal.

Following SV fusion, the *cis*-SNARE complex in the plasma membrane is disassembled by the combined actions of NEM-sensitive factor and soluble NSF-attachment proteins 9. SybII is therefore now free in the same membrane as its t-SNARE partners SNAP-25 and syntaxin. It would be highly favourable if sybII was prevented from reforming *cis*-SNARE complexes, since these complexes are extremely stable with a very low rate of spontaneous dissociation 62. One potential role for synaptophysin (which is also now in the plasma membrane) would be to prevent formation of ectopic *cis*-SNARE complexes, allowing t-SNAREs to remain available to receive fusogenic SV-located sybII. Although synaptophysin is displaced from sybII by t-SNARES when in SVs 39, it may still prevent sybII entry when the SNAREs are in the same membrane (i.e. after fusion).

An additional benefit of the prevention of *cis*-SNARE complexes in the plasma membrane by synaptophysin is that it should maintain monomeric sybII in a preferred conformation for recognition by the endocytosis machinery (Figure 2). Since synaptophysin interacts with sybII via their transmembrane domains, this should allow access of the ANTH domain of AP-180/CALM to the exposed SNARE motif. Thus synaptophysin presents sybII to AP-180 in a ‘retrieval competent’ conformation. Synaptophysin may also facilitate sybII retrieval by recruiting other endocytic machinery such as the AP-2 complex via canonical tyrosine motifs on its cytosolic C-terminus. The recruitment of AP-2 would increase the avidity of this plasma membrane sybII–synaptophysin complex further, since AP-2 and AP-180 share a well characterized interaction which catalyses the assembly of the clathrin coat 63. Furthermore a priming interaction of the ANTH domain with clathrin may also facilitate access to the sybII SNARE motif 64. Thus via a series of coordinated interactions, synaptophysin and AP-180 may work together to efficiently cluster and sort sybII to the nascent SV during endocytosis.

This working model has close parallels with the recognition and retrieval of other SV cargo; regardless of whether they contain canonical endocytic motifs. For example the glutamate transporter v-GLUT1 and the calcium sensor synaptotagmin I both contain classical adaptor protein complex recognition motifs 65,66, however, they still require endophilin 3 and stonin 2 4 to facilitate their respective retrieval. In a further parallel, the transmembrane SV protein SV2A is also required for efficient retrieval of synaptotagmin I from the plasma membrane 6. SV2A contains canonical AP-2 interaction motifs, the mutation of which perturbs the retrieval of both itself and synaptotagmin I. It is therefore temping to speculate that the model proposed above may be able to be applied more generally in terms of SV cargo retrieval.

## Mechanism of sybII Trafficking

The proposed model suggests a key role for synaptophysin in the trafficking of sybII during SV endocytosis, however, very little is known about the molecular basis of this function. Some clues have been provided via synaptophysin mutants identified in XLID patients 67. Two independent mutants that were truncated prior to their second transmembrane domain could not rescue sybII retrieval in knockout neurons 54. Moreover they were dominant-negative when expressed in wild-type neurons, such that their overexpression phenocopied synaptophysin knockout for both sybII localization and retrieval 54. Interestingly these mutants were toxic when expressed in the absence of exogenous sybII, suggesting that this first transmembrane domain forms at least part of the interaction platform between their transmembrane domains.

Two other XLID synaptophysin mutants were unable to fully rescue sybII retrieval in synaptophysin knockout cells 54. One contained a point mutation in the fourth transmembrane domain (G217R) hinting at potentially defective transmembrane interactions. The other mutant contained 156 novel amino acids due to a frame shift in its C-terminus. The inability of this mutant to effectively retrieve sybII suggests that the synaptophysin C-terminus may facilitate sybII retrieval, potentially via the recruitment of AP-2, which has been shown to bind to the isolated synaptophysin C-terminus (M. Cousin, unpublished observations). In support, C-terminally truncated synaptophysin does not rescue surface-trapped sybII in a heterologous expression system 33. A recent study has shown that exogenously expressed synaptophysin encompassing a C-terminal truncation retrieved with normal kinetics in cultured neurons 42, suggesting a potential disconnect between synaptophysin and sybII retrieval during SV endocytosis. Therefore it will be critical to systematically examine the role of the synaptophysin C-terminus in sybII retrieval at the earliest opportunity.

## Trafficking of Non-canonical sybs by Sybtraps

Multiple homologues of syb exist on SVs 7,68. These non-canonical sybs control the fusion of functionally distinct SVs that mediate either spontaneous or asynchronous neurotransmitter release 69–71. For example both sybVII and Vps10p-tail-interactor-1a (Vti1a) are proposed to define the resting pool of SVs 69,70. The resting SV pool is refractory to stimulation and is proposed to be the pool responsible for spontaneous release 69,70,72. In contrast sybIV drives asynchronous release evoked by strong stimulation 71. These non-canonical sybs have specific patterns of trafficking that are distinct from sybII. SybVII and Vti1a are reluctantly trafficked upon stimulation, however, they readily visit the plasma membrane during spontaneous SV recycling 69,70. Alternatively, sybIV displays an evoked retrieval during stimulation and then a post-stimulation insertion into the plasma membrane 71. These non-canonical sybs share similar SNARE and transmembrane domains to sybII, but in addition sybVII and Vti1a have an extended N-terminal region termed a longin domain 73. The longin domain of sybVII interacts with both the auxiliary adaptor Hrb and the adaptor protein complex AP-3 74–76. Furthermore sybIV contains a canonical dileucine motif allowing it to be recognized by adaptor protein complexes 77. Multiple non-canonical sybs interact with CALM via their SNARE motif 64 and removing either of the canonical endocytosis interaction interfaces from sybVII and sybIV converted them to being dependent on CALM for their internalization 78. Thus the reliance of known Sybtraps for retrieval of non-canonical sybs raises intriguing questions regarding the potential role of synaptophysin and α-synuclein in both their trafficking and biological function.

## Sybtrap Dysfunction: A Harbinger of Disease

SybII is crucial for neuronal function and its loss is incompatible with life 49, thus the efficient trafficking and chaperoning of sybII by Sybtraps is essential for the maintenance of human health. As discussed above four distinct mutations in the gene encoding synaptophysin were identified in families with XLID 67. All of these mutants were defective for sybII retrieval 54, suggesting that efficient trafficking of sybII during SV endocytosis is critical for the maintenance of synaptic health. In agreement synaptophysin knockout mice display an intellectual disability phenotype, with hyperactive behaviour and parallel defects in learning and memory 79. Perturbation of synaptophysin-dependent sybII traffic may also contribute towards psychiatric, as well as neurodevelopmental disorders. Schizophrenia is a relevant example, where reduced levels of synaptophysin are linked to symptoms and a series of mutants have been identified in patients displaying the disorder 80,81.

Efficient sybII trafficking is likely to be essential for maintaining neuronal health over an individual's lifetime, with small cumulative deficits in sybII traffic potentially culminating in a neurodegenerative phenotype. The Sybtrap α-synuclein has a long-identified role in the pathogenesis of Parkinson's Disease 57. One would predict that a progressive decrease in the efficiency of sybII chaperoning in later life would result in more misfolded forms of the protein and a decrease in synaptic efficiency. In support of this, αβγ-synuclein triple knockout mice display an age-dependent decrease in SNARE complex assembly that is associated with impaired survival 58. Parkinson's Disease-associated mutant variants of α-synuclein generally have the same capacity to bind to sybII and catalyse SNARE complex assembly 59, suggesting this is not the primary cause of their effect. However, their propensity to aggregate suggests that lower levels of soluble (and presumably functional) α-synuclein would be present *in vivo* to perform normal neuronal roles in facilitating sybII function with increasing age.

The potential long-term consequences of ineffective sybII trafficking may contribute to other neurodegenerative conditions such as Alzheimer's disease. For example, the toxic amyloid-β peptide (Aβ42), which is implicated in Alzheimer's disease pathogenesis, interferes with the synaptophysin-sybII interaction 82, suggesting that disruption of efficient sybII retrieval may contribute to disease pathogenesis. Furthermore, recent studies have suggested that Aβ42 may impact directly on SV endocytosis 83,84. Mutations in the gene encoding the Sybtrap CALM were identified to be associated with a risk of Alzheimer's Disease in a genome-wide associated study 85. Recent studies identified CALM as a suppressor of Aβ42-mediated toxicity in both yeast and *C. elegans*, and it also protected cultured mammalian neurons from Aβ oligomers 86. How this potentially links to sybII traffic is still undetermined but should be an area for urgent investigation.

The two most abundant proteins on the SV, sybII and synaptophysin have received divergent degrees of attention over the past 30 years. The key role played by sybII in evoked neurotransmitter release has lead to the intense study of its molecular mode of action. Conversely after initial studies showing little overt phenotype in knockout mice, research into synaptophysin waned for a number of years. The discovery that synaptophysin belonged to a growing class of sybII trafficking partners, the Sybtraps, has revitalized research into its function and in doing so have revealed the critical role sybtraps perform in ensuring sybII always makes it to the correct place, at the correct time and in the correct position.
